# Docosahexaenoic Acid Increases the Potency of Soluble Epoxide Hydrolase Inhibitor in Alleviating Streptozotocin-Induced Alzheimer’s Disease-Like Complications of Diabetes

**DOI:** 10.3389/fphar.2019.00288

**Published:** 2019-04-24

**Authors:** Rohit Pardeshi, Nityanand Bolshette, Kundlik Gadhave, Mohammad Arfeen, Sahabuddin Ahmed, Rohitash Jamwal, Bruce D. Hammock, Mangala Lahkar, Sumanta Kumar Goswami

**Affiliations:** ^1^Department of Biotechnology, National Institute of Pharmaceutical Education and Research (NIPER), Gauhati Medical College and Hospital, Guwahati, India; ^2^Institutional Level Biotech Hub (IBT Hub), Department of Biotechnology, National Institute of Pharmaceutical Education and Research (NIPER), Gauhati Medical College and Hospital, Guwahati, India; ^3^School of Basic Sciences, Indian Institute of Technology Mandi, Kamand, India; ^4^Laboratory of Neurobiology, Department of Pharmacology and Toxicology, National Institute of Pharmaceutical Education and Research (NIPER), Gauhati Medical College and Hospital, Guwahati, India; ^5^Biomedical and Pharmaceutical Sciences, The University of Rhode Island, Kingston, RI, United States; ^6^Hammock Laboratory of Pesticide Biotechnology, Department of Entomology and Nematology, and Comprehensive Cancer Center, University of California, Davis, Davis, CA, United States

**Keywords:** Alzheimer’s disease-like complications of diabetes, sEH inhibitor TPPU, DHA, Morris water maze, novel object recognition test, inflammatory cytokines

## Abstract

Diabetes is a risk factor for Alzheimer’s disease and it is associated with significant memory loss. In the present study, we hypothesized that the soluble epoxide hydrolase (sEH) inhibitor N-[1-(1-oxopropyl)-4-piperidinyl]-N’-[4-(trifluoromethoxy)phenyl)-urea (also known as TPPU) could alleviate diabetes-aggravated Alzheimer’s disease-like symptoms by improving memory and cognition, and reducing the oxidative stress and inflammation associated with this condition. Also, we evaluated the effect of edaravone, an antioxidant on diabetes-induced Alzheimer’s-like complications and the additive effect of docosahexaenoic acid (DHA) on the efficacy of TPPU. Diabetes was induced in male Sprague-Dawley rats by intraperitoneally administering streptozotocin (STZ). Six weeks after induction of diabetes, animals were either treated with vehicle, edaravone (3 or 10 mg/kg), TPPU (1 mg/kg) or TPPU (1 mg/kg) + DHA (100 mg/kg) for 2 weeks. The results demonstrate that the treatments increased the memory response of diabetic rats, in comparison to untreated diabetic rats. Indeed, DHA + TPPU were more effective than TPPU alone in reducing the symptoms monitored. All drug treatments reduced oxidative stress and minimized inflammation in the brain of diabetic rats. Expression of the amyloid precursor protein (APP) was increased in the brain of diabetic rats. Treatment with edaravone (10 mg/kg), TPPU or TPPU + DHA minimized the level of APP. The activity of acetylcholinesterase (AChE) which metabolizes acetylcholine was increased in the brain of diabetic rats. All the treatments except edaravone (3 mg/kg) were effective in decreasing the activity of AChE and TPPU + DHA was more efficacious than TPPU alone. Intriguingly, the histological changes in hippocampus after treatment with TPPU + DHA showed significant protection of neurons against STZ-induced neuronal damage. Overall, we found that DHA improved the efficacy of TPPU in increasing neuronal survival and memory, decreasing oxidative stress and inflammation possibly by stabilizing anti-inflammatory and neuroprotective epoxides of DHA. In the future, further evaluating the detailed mechanisms of action of sEH inhibitor and DHA could help to develop a strategy for the management of Alzheimer’s-like complications in diabetes.

## Introduction

Diabetes mellitus (DM) is a complex metabolic disorder which adversely affects multiple physiological systems including the central nervous system and cardiovascular system, and the disease is associated with altered memory function, cardiac stress, endothelial dysfunction and neuropathy (Islam et al., 2017; Bhadri et al., 2018; Minaz et al., 2018). Both type 1 and type 2 diabetes induce changes in the neurones, brain and memory as seen in the case of Alzheimer’s disease (Zhao and Townsend, 2009; Ouwens et al., 2014; Rdzak and Abdelghany, 2014; King et al., 2015; Meneilly and Tessier, 2016; Morales-Corraliza et al., 2016; Chen et al., 2018; Pappas et al., 2018; Takeuchi et al., 2018; Yashkin et al., 2018).

Alzheimer’s disease (AD) is the leading cause of dementia. Characterized by memory and cognitive impairment in aging populations, it poses a significant health challenge. Although, AD is reported with significant neurodegeneration by accumulation of amyloid beta (Aβ) and tau protein aggregates in the human brain (Kolarova et al., 2012; Peraza et al., 2016; Kim et al., 2018), its exact potential cause is still not completely understood (Gadhave et al., 2016). Recently, the World’s Alzheimer’s report 2018 estimated that worldwide around 50 million people are living with dementia and this number is set to reach 82 million by 2030, and more than triple to 152 million by 2050. Moreover, the total estimated worldwide cost is USD 1 trillion by 2018, and that will rise to USD 2 trillion by 2030 (Alzheimer’s Disease International, 2018). Interestingly, impaired insulin signaling is linked to memory loss.

Insulin signaling is crucial for synaptogenesis, neuronal development, and survival (Apostolatos et al., 2012). The activation of GSK-3β, inflammation, oxidative stress, and altered glucose metabolism are critical features of both Type II DM and AD (De Felice et al., 2014; Bedse et al., 2015; Pardeshi et al., 2017). Importantly, proinflammatory signaling, oxidative stress, and prolonged metabolic stress contribute to insulin resistance (Gregor and Hotamisligil, 2011; Henriksen et al., 2011). Cognitive defects in diabetes include altered psychomotor efficiency, attention, learning memory, and mental flexibility (Bakris et al., 2009). In addition to regulation of blood glucose, insulin also modulates synaptic plasticity, cognition and aging-related neurodegeneration (Zhao and Townsend, 2009). Increase in the level of blood glucose and hyperinsulinemia are known to cause neuronal death followed by neurodegeneration as seen with AD (Zhao and Townsend, 2009). Additionally, the homeostasis of glucose and lipid affect the production and clearance of Aβ, phosphorylation of tau and neurodegeneration (Sato and Morishita, 2015). Insulin resistance was found to be associated with significantly lower regional cerebral glucose metabolism and impaired memory performance (Willette et al., 2015). Though insulin resistance and deficiency augment the phosphorylation of Aβ and tau and promote the development of AD (Garwood et al., 2015), the pathology of the process remains incompletely understood (Bedse et al., 2015). DM might facilitate the development of AD through an increase in the level of oxidative stress and advanced glycation end products (AGEs), which causes structural and functional abnormalities in the brain (Gispen and Biessels, 2000; Biessels et al., 2002).

The involvement of soluble epoxide hydrolase in vascular cognitive decline has recently been reported (Nelson et al., 2014). It was found that a soluble epoxide hydrolase inhibitor (sEHI) alleviates transmissible spongiform encephalopathies (TSEs) associated with neuronal loss and inflammation (Poli et al., 2013). Earlier, we demonstrated that the sEHI, TPPU, minimizes memory impairment in diabetic rats (Minaz et al., 2018). These lines of research suggest that sEHIs may have potential therapeutic effect in managing AD associated memory impairment. We have previously demonstrated the anti-inflammatory property (Goswami et al., 2016, 2017) of TPPU in addition to its ability to reduce oxidative stress (Goswami et al., 2016) including the diabetes-induced oxidative stress in the brain (Minaz et al., 2018), but it has not been evaluated in AD-like condition. We hypothesized that TPPU could alleviate diabetes-induced Alzheimer-like complexities by decreasing the oxidative stress and inflammation. Primarily, sEH inhibitors exert anti-inflammatory activity by preserving the levels of both ω-6 and ω-3 epoxy fatty acids including epoxyeicosatrienoic acids (EETs) which are metabolized by sEH (Morisseau and Hammock, 2013). In general, epoxy fatty acids produced from DHA, an ω-3 fatty acid are more potent anti-inflammatory agents than arachidonic acid (ω-6) derived EETs in several different bioassays. Therefore, we assessed the effect of DHA on the anti-Alzheimer activity of TPPU. For comparative analysis, we have evaluated the effect of edaravone which is known to possess anti-oxidant and anti-inflammatory properties (Watanabe et al., 2008) in diabetes-induced Alzheimer’s-like complexities.

## Materials and Methods

### Chemical and Reagents

TPPU was synthesized in the Hammock Laboratory of Pesticide Biotechnology, University of California, Davis, the United States as described earlier (Rose et al., 2010). DHA (TCI chemicals, United States), PEG400 (Sigma-Aldrich, United States), 3-methyl-1-phenyl-5-pyrazolone/edaravone (TCI chemicals, United States), SD Check blood glucose meter (SD Biosensor Inc., South Korea), ELISA kit for cytokine estimation (Elabscience Biotechnology Co., Ltd., Wuhan, China), HiPuraA^TM^ Total RNA Miniprep purification kit (Himedia, India), primers (Integrated DNA Technologies, United States) were collected. All other reagents were of analytical grade.

### Animal Studies

Thirty-six male Sprague-Dawley rats, 6–8 weeks old, between 200 and 250 g were used. The animals were randomized so that each group had animals of similar weight and age. Animals were maintained under standard laboratory conditions. The animal experiment protocols were approved (MC/05/2015/73) by the Institutional Animal Ethics Committee (IAEC), Gauhati Medical College and Hospital, India *(CPCSEARegistration No. 351; 3/1/2001)*. Diabetes was induced by administration of streptozotocin (50 mg/kg body weight, i.p.) dissolved in 0.1 M cold citrate buffer (pH 4.5). Blood was collected from the tail vein, and blood glucose was quantified using SD Check blood glucose meter 48 h after STZ administration (SD Biosensor Inc., South Korea). Only animals with a blood glucose level > 250 mg/dl were used in the study. The day of diabetes assessment was considered as day 1.

**FIGURE 1:**
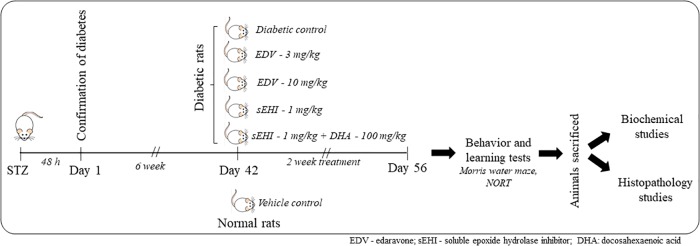
A total number of 36 rats were included in the study containing 6 rats in 6 groups. One group was maintained as a vehicle control group, and another 5 groups of rats were dosed with STZ. Induction of diabetes was confirmed after 48 h. Animals were maintained for six weeks under the diabetic condition for the development of memory impairment. Then animals were dosed for two weeks with edaravone, sEH inhibitor TPPU or DHA + TPPU. Memory function was evaluated on the 56th day, and animals were sacrificed. Biochemical tests and histopathology were performed to evaluate the protective effect of treatments.

Diabetic rats were randomly distributed to five groups and treated as described in Figure 1. Non-diabetic animals (not treated with STZ) were used as normal control whereas diabetic rats treated with the vehicle was used as positive/diabetic control. Diabetic rats were administered edaravone/EDV (3 mg/kg), edaravone (10 mg/kg), TPPU (1 mg/kg) or TPPU + DHA (100 mg/kg). The dose of TPPU was selected based on our previous studies on anti-inflammatory and memory protective activity of the enzyme inhibitor (Goswami et al., 2016; Minaz et al., 2018). Epoxy fatty acids generated from DHA were more stable and potent in the presence of the sEHI. Therefore, DHA was selected based on previous reports (Ulu et al., 2013, 2014). The dose of DHA or it’s derivative to ameliorate Alzheimer’s disease is 40–200 mg/kg in animal models (Cansev et al., 2008; Sugasini et al., 2017). We have selected 100 mg/kg dose of DHA for this study. The dose of edaravone was selected based on published data on its protective effect on diabetes-induced cognitive impairment (Zhou et al., 2013; Reeta et al., 2017). Two doses of edaravone were selected to evaluate if the anti-oxidant exert a dose-dependent effect.

The treatments were administered for 6 weeks after STZ injection and dosing were continued for two weeks. TPPU was dissolved in PEG400 and DHA in 5% of acacia solution. Weekly fasting blood glucose was measured by using SD Check blood glucose meter. On the day of the last dose, the blood was collected from animals through the tail vein for glucose analysis. Further, animals were sacrificed to isolate the hippocampus as well as remaining parts of the brain to perform subsequent biochemical and histopathological studies (Figure 1).

### Behavioral and Learning Tests

The Morris water maze (MWM) test was used to assess the learning and memory function (Vorhees and Williams, 2006). Novel object recognition test (NORT) was performed to evaluate recognition memory as described previously (Singla and Dhawan, 2017).

For conducting the MWM test, a circular tank consisting of diameter 120 cm and a height of 50 cm were used. The MWM had four equal quadrants containing non-transparent water at room temperature (24–26°C). The water level was high enough to prevent the animal from touching the base of the tank and low enough to prevent animal from jumping out of the water. A platform measuring 10 cm, on which a rat can stand/sit, was used to assess memory function. The rats were trained for learning to locate the visible platform during the acquisition phase of training, then the memory of rats was evaluated during the evaluation phase by allowing them to discover the hidden platform. During the acquisition phase, the platform was placed 1 cm above the water level so that the rats can quickly identify the platform and sit on it when forced to explore the tank for 2 min. In case the animals failed to locate the platform, the researcher placed them on the platform for half a minute during five days of training. Daily four trials were run for each rat individually with a 5 min interval between each session in a day. During each test, the rats were placed in different quadrants. To analyze the retention of memory, the animals were positioned in the quadrant with their head facing the wall and allowed to explore the hidden platform 1 cm below the surface to avoid drowning. Retention latencies were recorded by measuring the time taken by animals to locate the hidden platform. Also, reference memories were evaluated by measuring the number of times each animal crossed over a reference point, where the platforms were kept during training phase after the animals were placed on a novel place in the quadrant. For calculating reference memory, no platforms were set during the test.

Novel object recognition test, a commonly used test, to evaluate the recognition memory were employed in this study. The ability of animals to identify the old object and to spend more time in exploring new object than the old object was considered as a marker of recognition memory. The test has 3 phases/sessions to quantify recognition index: *habituation phase* where the animals were familiarized with an open field box (36 × 50 × 36 cm), *familiarization phase* where the animals were acquainted with two objects, and finally a *test phase* where the animals were subjected to a novel object. First, the animals were habituated to a black open field box for 5 min, two times per day for a total duration of three days. Then, the animals were familiarized with a rectangular wooden block and a rubber ball in the open field box and allowed to explore for 10 min after placing them with their heads facing opposite to objects. Next day, the animals were subjects to a new and novel object, i.e., a small wooden box along with two old objects for 3 min in the open field box. Time spent on exploring the objects was recorded. Recognition index was calculated by dividing the time spent by animals in exploring known objects from the time spent in exploring novel object and presented as a percentage. The total exploratory period was also measured to validate that the recognition index calculated was not due to activation or inactivation of sensorimotor function. During the whole experiment, the open field box and objects were cleaned with 70% ethanol regularly for each animal and each session to avoid olfactory cues.

### Tissue Collection, Preparation and Histopathology Studies

Animals were sacrificed by cervical dislocation under anesthesia and brains were collected. One half of the brain including hippocampus was fixed in 10% buffered formalin. A small portion of the hippocampus was used to isolate RNA. Another half of the brain was dipped in liquid nitrogen for 30 s and later stored at −80°C until further use. A portion of each brain was homogenized (1:9, tissue: buffer, w/v) in ice-cold phosphate buffer saline (0.05 M, pH 7.0) (Heidoph, Silent Crusher S, Germany). The homogenate was centrifuged at 10000 *g* for 30 min at 4°C and supernatant for each tissue was collected. Supernatants were subsequently used for biochemical characterization as described below. Formalin-fixed tissue was stained with eosin using previously described procedures (Fischer et al., 2008).

### Estimation of Oxidative Stress

The concentration of MDA in hippocampal homogenates as an index of lipid peroxidation was determined according to a previously described method (Ohkawa et al., 1979). Nitric oxide content in the hippocampus was determined indirectly by measuring the level of nitrite (Green et al., 1982). Glutathione concentration in the hippocampus was measured using reduced glutathione (GSH) as the reference standard as described by Ellman (1959). Total protein was estimated using Lowry’s method (Waterborg and Matthews, 1984).

### Quantification of Inflammatory Markers (IL-1β, IL-6, and IL-10)

The levels of pro-inflammatory cytokines (IL-1β and IL-6) and anti-inflammatory cytokine (IL-10) in hippocampus tissues of control and treatment groups were determined by ELISA (Elabscience Biotechnology Co. Ltd., Wuhan, China).

### Gene Expression Study and Enzyme Activity

Total RNA was isolated from the freshly collected hippocampal region of the brain of mice using HiPuraA^TM^ Total RNA Miniprep purification kit (Himedia, India). The assay was performed according to kit protocol, and NCBI Primer-BLAST Tools designed primers were used. The PCR products were resolved on 1.7% agarose gel. Gel image was taken by UVI TEC Essential V4 Gel Doc XR^+^system, and image analysis was performed with the help of Image lab-5.1 gel analysis software. See the data in Table 1. The activity of AChE was determined using acetylcholinesterase assay kit from Abcam.

**Table 1:** PCR product size and Tm of APP and β-Actin from rat.

Sr.No	Gene (Species: *Rattus norvegicus*)	PCR Product size	Tm
1	Amyloid Precursor Protein (APP)	90 bp	59.0°C
	forward primer: GCAATGATCTCCCGCTGGTA;		
	reverse primer: AAAGTTGTTCCTGTTGCCGC		
2	Beta Actin (β-Actin)	76 bp	60.0°C
	forward primer: TCTGAACCCTAAGGCCAACC;		
	reverse primer: TACGTACATGGCTGGGGTGT		

### Histopathology

At the end of the study, the brain of the rat was removed after cervical dislocation and immediately fixed in 10% buffered formalin. H&E staining was performed using a standardized protocol (Bancroft and Gamble, 2008; Amin et al., 2013).

### Statistical Analysis

All values are expressed as means ± SEM and were analyzed using one-way analysis of variance (ANOVA) followed by Tukey’s method for *post hoc* analysis. All the statistical analysis were performed on Prism 6 and *P* < 0.05 was considered statistically significant (GraphPad Inc., United States).

## Results

### TPPU and TPPU + DHA Treatment Does Not Decrease the Level of Glucose in Serum

As discussed earlier, six weeks after the induction of diabetes, the animals were treated with curative doses of edaravone, TPPU or TPPU + DHA for two weeks. The treatment did not affect the level of glucose in the serum in comparison to diabetic control animals. The serum glucose levels of diabetic control and diabetic treated animals ranged from 400 to 450 mg/dL whereas the serum glucose level of normal healthy rats was around 90 to 100 mg/dL.

### TPPU and TPPU + DHA Alleviate Memory Dysfunction in Diabetes

**FIGURE 2:**
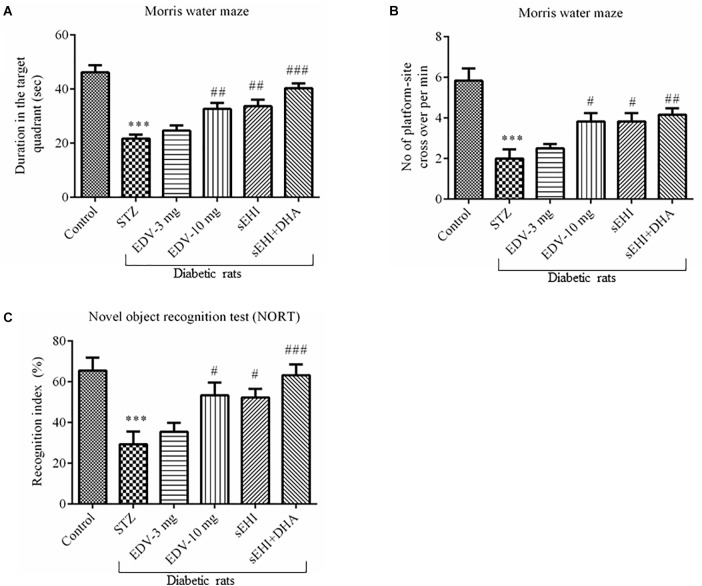
TPPU + DHA decreases the degree of memory impairment in diabetic rats. Memory function was assessed by quantifying the **(A)** duration spent in target quadrant of the MWM, **(B)** a number of platform site crossover per minutes in MWM, and **(C)** recognition index. Memory impairment was observed in diabetic rats in comparison to normal healthy rats. Treatment with edaravone (only 10 mg/kg), TPPU or TPPU + DHA alleviated the memory impairment in diabetic rats. The data are presented as mean ± SEM of 6 readings. One-way ANOVA followed by Tukey’s multiple comparison test was used for statistical analysis. ^∗∗∗^*p* < 0.001 when compared with healthy rats; *N* = 6, ^#^*p* < 0.05, ^##^*p* < 0.01, ^###^*p* < 0.001 when compared with diabetic control rats.

Effect of TPPU on memory function as assessed from the Morris water maze test is summarized in Figure 2. In the present study, the AD-like complication was induced by administration of STZ (50 mg/kg i.p.) and confirmed by a significant decrease in “sporadic memory” and “recognition memory” in diabetic rats in comparison to healthy control animals.

Diabetic control rats spent less time (22 ± 2 s) in the target quadrant when compared with healthy vehicle control rats (46 ± 3 s). Rats treated with TPPU spent more time (33 ± 2 s) in the target quadrant of the water maze which is a result of development in sporadic memory. DHA significantly potentiated the effect of TPPU on memory function because the rats treated with DHA + TPPU spent more time in the maze (40 ± 2 s). Edaravone at higher concentration also increased sporadic memory of diabetic rats because rats spent more time in the maze (33 ± 2; Figure 2A). The number of times the diabetic rats (2 ± 0.4) crossed platform-site was less, in comparison to vehicle control rats (5.8 ± 0.6). Diabetic rats treated with edaravone (only 10 mg/kg) (3.8 ± 0.4), TPPU (3.8 ± 0.4), or DHA + TPPU (4.2 ± 0.3) significantly crossed more lines, in comparison to diabetic rats indicating an increase in the sporadic memory in the treated animals. All treatment groups showed similar results (Figure 2B).

A significant decrease in the recognition index was observed in diabetic rats (29 ± 6%) in comparison to normal control rats (65 ± 6%) indicating a decrease in recognition memory. An increase in the percent of recognition index in the treatment group of edaravone (only 10 mg/kg) (53 ± 6%), TPPU (52 ± 4%) and sEHI + DHA (63 ± 5%) were observed in comparison to diabetic control rats. Thus, the treatment groups showed an improvement in sporadic memory, recognition memory, and learning memory (Figure 2C). One-way ANOVA followed by Tukey’s multiple comparison test were used for calculating statistical significance. Values were presented as mean ± SEM of 6 observations.

### DHA + TPPU Minimize Oxidative Stress

**FIGURE 3:**
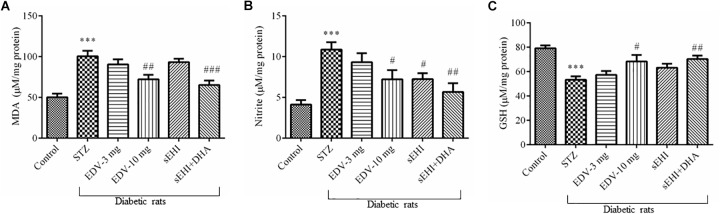
TPPU + DHA decreases oxidative stress in the hippocampus of diabetic rats. Oxidative stress was evaluated by quantifying an increase in the levels of **(A)** MDA and **(B)** nitrite but a decrease in the level of **(C)** GSH. Oxidative stress was increased in the brain of diabetic rats, in comparison to normal control rats. Treatment with edaravone (10 mg/kg), TPPU or TPPU + DHA minimized the oxidative stress in the brain of diabetic rats, in comparison to diabetic control rats. The data are presented as mean ± SEM of 6 readings. One-way ANOVA followed by Tukey’s multiple comparison test was used for statistical analysis. ^∗∗∗^*p* < 0.001 when compared with healthy rats; *N* = 6, ^#^*p* < 0.05, ^##^*p* < 0.01, ^###^*p* < 0.001 when compared with diabetic control rats.

An increase in the level of MDA (100 ± 7 μM/mg protein) was observed in the diabetic rats in comparison to vehicle control rats (50 ± 5 μM/mg protein) suggesting an increase in the oxidative stress, whereas treatment with edaravone (10 mg/kg) (72 ± 5 μM/mg protein) or TPPU + DHA (65 ± 5 μM/mg protein) significantly minimized an increase in the level of MDA (Figure 3A). The level of nitrite in the diabetic control group (10.8 ± 0.9 μM/mg protein) significantly increased when compared with vehicle group (4.1 ± 0.5 μM/mg protein) indicating an increase in the oxidative stress in diabetic rats. Edaravone (10 mg/kg) (7.2 ± 1 μM/mg protein) and TPPU (7.2 ± 0.7 μM/mg protein) prevented an increase in the level of nitrite. The combination of DHA and TPPU (5.7 ± 1 μM/mg protein) was more effective than TPPU alone in reducing oxidative stress in terms of decreasing the level of nitrite (Figure 3B). In diabetic rats (53 ± 3 μM/mg protein), a significant decrease in the level of endogenous antioxidant GSH was observed when compared with normal control rats (79 ± 2 μM/mg protein). Treatment of edaravone (10 mg/kg) (68 ± 5 μM/mg protein) significantly prevented an increase in the level of GSH in diabetic rats, and when the sEHI was treated along with DHA (70 ± 3 μM/mg protein) the level of GSH was increased higher than the diabetic control (Figure 3C). One-way ANOVA followed by Tukey’s multiple tests were used for calculating statistical significance. Values are presented as mean ± SEM of 6 observations.

### TPPU and DHA + TPPU Decrease Inflammation in the Hippocampus of Diabetic Rats

**FIGURE 4:**
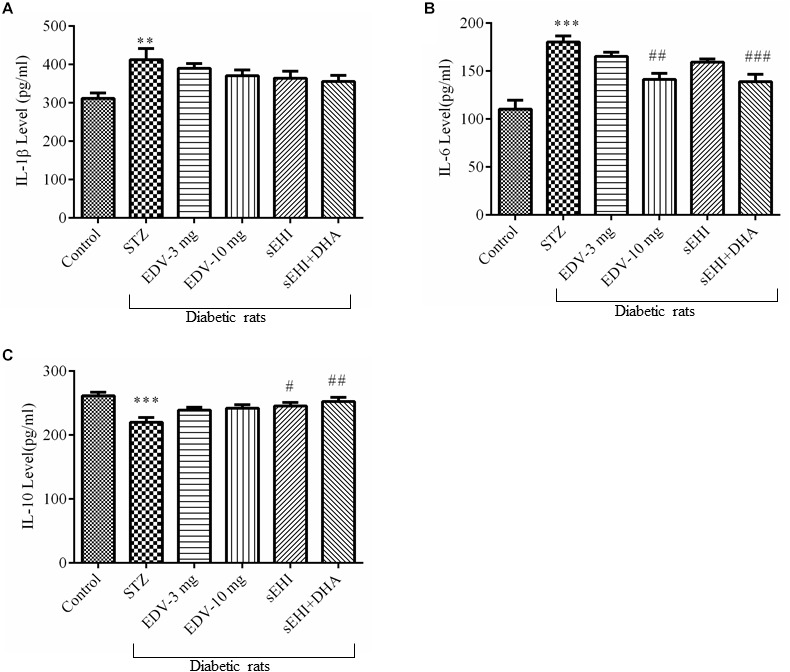
TPPU + DHA decreases the level of neuroinflammation. **(A)** An increase in the level of IL-1β was observed in the hippocampus of diabetic rats in comparison to normal rats. None of the treatments decreased the level of IL-1β. **(B)** The level of IL-6 was increased in the hippocampus of diabetic rats, in comparison to normal rats. Treatment with either edaravone (10 mg/kg) or TPPU + DHA decreased the level of IL-6 in the diabetic rats. **(C)** The level of IL-10 was decreased in the brain of diabetic rats, in comparison to normal rats. TPPU increased the level of IL-10 in diabetic rats, and DHA potentiated the efficacy of TPPU in increasing the level of IL-10. The data are presented as mean ± SEM of 6 readings. One-way ANOVA followed by Tukey’s multiple comparison test was used for statistical analysis. ^∗∗^*p* < 0.01 and ^∗∗∗^*p* < 0.001 when compared with healthy rats; *N* = 6, ^#^*p* < 0.05, ^##^*p* < 0.01, ^###^*p* < 0.001 when compared with diabetic control rats.

An increase in the neuroinflammation was evident in the hippocampus of diabetic rats due to the rise in the level of pro-inflammatory marker IL-1β (Figure 4A) (412 ± 29 pg/ml) and IL-6 (Figure 4B) (180 ± 6 pg/ml) and decrease in the level of anti-inflammatory marker IL-10 (Figure 4C) (220 ± 7 pg/ml) in comparison to the healthy normal rats (IL-1β, 311 ± 14 pg/ml; IL-6, 110 ± 9 pg/ml; IL-10, 261 ± 5 pg/ml). None of the treatments had any effect on the level of IL-1β, but treatment with edaravone (10 mg/kg) (141 ± 6 pg/ml) or TPPU + DHA (139 ± 7 pg/ml) prevented an increase the level of IL-6. TPPU (245 ± 6 pg/ml) or TPPU + DHA (252 ± 6 pg/ml) prevented a decrease in the level of IL-10. Overall, the treatments decreased the degree of inflammation in diabetic rats in comparison to diabetic control rats. One-way ANOVA followed by Tukey’s multiple tests were used for calculating statistical significance. Values are presented as mean ± SEM of 6 observations.

### DHA + TPPU Decreases the Expression of APP and Activity of AChE

**FIGURE 5:**
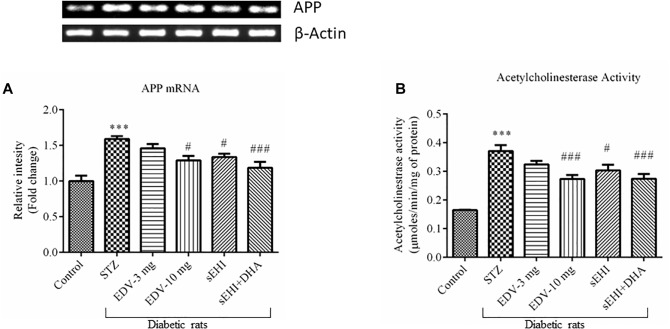
DHA + TPPU decreases the expression of APP **(A)** and activity of AChE **(B)** in the hippocampus of diabetic rats. TPPU and both TPPU + DHA decreased the markers of Alzheimer’s disease in diabetic rats. Expressions of APP and activity of AChE were quantified in the brains of normal healthy rats, diabetic rats and diabetic rats treated with either edaravone, TPPU or TPPU + DHA. The level of APP and activity of AChE were increased in the brains of diabetic rats, in comparison to normal healthy rats. Treatment with edaravone (10 mg/kg), TPPU or TPPU + DHA prevented an increase in the levels of APP, in comparison to diabetic control rats. Similarly, treatment with edaravone (10 mg/kg), TPPU or TPPU + DHA treatment minimized an increase in the activity of AChE. The data are presented as mean ± SEM of 6 readings. One-way ANOVA followed by Tukey’s multiple comparison test was used for statistical analysis. ^∗∗∗^*p* < 0.001 when compared with healthy rats; *N* = 6, ^#^*p* < 0.05, ^##^*p* < 0.01, ^###^*p* < 0.001 when compared with diabetic control rats.

The gene expression experimental data showed an increase in the expression of the APP gene, which is encoded for amyloid beta synthesis. Administration of STZ leads to overexpression of the APP mRNA (1.6 ± 0.0 fold) in diabetic rats, in comparison to vehicle control rats. The treatment with edaravone (10 mg/kg) (1.3 ± 0.1 fold) or TPPU (1.3 ± 0.0 fold) significantly decreased the expression of APP as compared to pathological control. DHA potentiated the effect of TPPU in decreasing (1.2 ± 0.1 fold) the level of APP (Figure 5A). TPPU minimized (*p* < 0.05) the level of mRNA of APP in diabetic rats, in comparison to diabetic control rats. DHA + TPPU was more effective in decreasing (*p* < 0.001) the level of mRNA of APP, in comparison to TPPU alone.

The activity of AChE was also increased (0.37 ± 0.02 μmoles/min/mg protein) in the STZ-treated group, in comparison to vehicle control rats (0.16 ± 0.00 μmoles/min/mg protein). AChE metabolizes ACh, and an increase in the activity of AChE would infer a reduction in the level of ACh, a neurotransmitter which supports cognitive function in cerebral cortex and hippocampus. Treatment with edaravone (10 mg/kg) (0.27 ± 0.01 μmoles/min/mg protein), TPPU (0.30 ± 0.02 μmoles/min/mg protein) or TPPU + DHA (0.27 ± 0.02 μmoles/min/mg protein) decreased the activity of AChE in diabetic rats, in comparison to diabetic control rats (Figure 5B). One-way ANOVA followed by Tukey’s multiple tests was used for calculating statistical significance. Values are presented as mean ± SEM of 6 observations.

### TPPU and TPPU + DHA Protect Hippocampal Neurons in Diabetes

**FIGURE 6:**
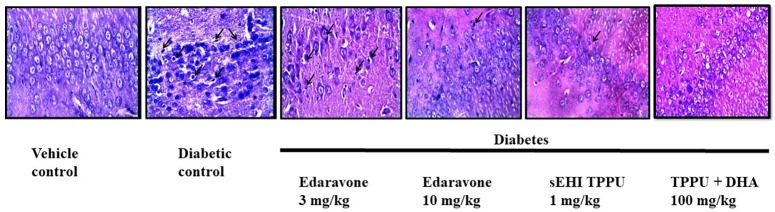
Edaravone, TPPU, and TPPU + DHA protect the pyramidal neurons in diabetes. In addition to quantifying the level of APP, we used H&E staining to study the histology of hippocampus. In comparison to healthy rats, pyramidal neurons in the hippocampus of diabetic rats were less in number, irregularly arranged and damaged which are characterized by pyknotic and indistinct nuclei. Treatment with a low dose of edaravone i.e., 3 mg/kg did not protect pyramidal neurons, but a high dose of edaravone i.e., 10 mg/kg protected the pyramidal neurons. As expected, the protection offered by TPPU was lesser than the combination of TPPU + DHA. *N* = 6 in each group. The images are presented as 400×.

H&E staining of the CA1 region of hippocampus from normal healthy rat revealed regularly arranged neurons with large, round, transparent, and intact nuclei. The pyramidal neurons in the CA1 region of the diabetic rat was severely damaged, disorderly arranged with significantly reduced in number, and characterized by pyknotic and indistinct nuclei which indicates neuron death. A decrease in the number of pyramidal neurons in the CA1 region of the hippocampus is reported in Alzheimer’s disease (Davies et al., 1992). Edaravone at 3 mg/kg failed to show any neuroprotection with characteristic pyknotic nuclei still visible. Treatment with edaravone at 10 mg/kg shows some neuroprotection where neurons are orderly arranged with still indistinct nuclei visible. Treatment with TPPU resulted in good neuroprotective activity. Treatment with sEHI + DHA resulted in better neuroprotective effect with a parallel decrease in hippocampal atrophy also to the visibility of prominent distinct nuclei (Figure 6). The qualitative neuroprotective effect of treatments was evaluated manually.

## Discussion

Diabetes, associated with insulin resistance and reduction in the level of insulin, mainly affects psychomotor activity, attention, learning, memory, cognition, mental flexibility in the brain (Bakris et al., 2009). Increased risk of dementia, including memory dysfunction associated with Alzheimer’s disease is prevalent among diabetic patients (Barbagallo and Dominguez, 2014). We observed a decrease in learning and memory of diabetic rats with a parallel increase in the level of inflammation, oxidative stress, APP and neuronal damage in comparison to normal healthy rats. Treatment with antioxidant (edaravone), sEHI (TPPU) or TPPU + DHA minimized diabetes-induced memory impairment, oxidative stress, and inflammation.

Additionally, the treatment also prevented an increase in the level of APP and neuronal damage. The up-regulation, trafficking, and dysregulation of amyloid precursor protein (APP) processing facilitate the Amyloid beta (Aβ) production that plays a vital role in AD pathogenesis (Wang et al., 2017). APP is an integral membrane glycoprotein that undergoes sequential proteolytic cleavage by β- and γ-secretase. Moreover, genomic duplication and mutations in APP can lead to alteration in its function and consequent Aβ metabolism (Dong et al., 2012). According to amyloid cascade hypothesis, the accumulation of Aβ peptide is the primary and sole event leading to the formation of senile plaques, then neurofibrillary tangles (NFTs), cell death, and eventually dementia (Armstrong, 2011). Senile plaques and NFTs are highly insoluble protein accumulations. Aβ triggers the conversion of tau from a normal to a toxic state. However, some pieces of evidence also show that toxic tau enhances Aβ toxicity via a feedback loop (Bloom, 2014). Aβ at normal physiological condition plays putative roles such as promoting recovery from injury, protecting the body from infections, regulating synaptic function (Brothers et al., 2018). But, Aβ acts as an initiator of progression of AD due to its overproduction or disturbed clearance, accumulation and aggregation (Sun et al., 2015). Tau is the microtubule-associated protein that regulates the assembly and structural stability of microtubules contributing to the proper function of the neuron. In AD, due to the hyperphosphorylation of tau it gets disassembles from microtubule and eventually, free tau gets to assemble and aggregates into paired helical filaments (Medeiros et al., 2011; Kolarova et al., 2012). The soluble forms of Aβ and tau peptides work together and independently accumulate into senile plaques and NFTs to produce neurodegeneration and synaptic loss (Bloom, 2014). Increase in Aβ 40 and Aβ 42 level has been found in the pathogenesis of AD (Findeis, 2007).

This study evaluated the effect of curative treatment of edaravone, TPPU or TPPU + DHA on diabetes-induced memory dysfunction. The work was conducted using 6-8-week-old male Sprague-Dawley rats supports our previous observation that inhibition of sEH in diabetic rats can protect memory function in 12-week-old male Wistar albino rats (Minaz et al., 2018). In the previous study, the increase in the memory function of diabetic rats was associated with a decrease in the levels of blood sugar after eight weeks of treatment with TPPU (0.1 and 0.3 m/kg) in comparison to diabetic control rats. The memory functions were assessed by using Barne’s maze and step-down test. Here, the memory function was evaluated by using Morris water maze and the novel object recognition test. Although both studies used different methods to assess memory function, we observed preservation of memory with the use of sEHI TPPU. In this study, the positive effect of TPPU on memory function was not associated with a decrease in the level of blood sugar which could be due to the shorter duration of treatment, i.e., two weeks. Improvement in memory function independent of the concentration of blood glucose suggests that the sEH inhibitor can exert its positive effect on memory directly by protecting neurons via decreasing inflammation and oxidative stress.

Diabetes-induced impairment of memory is associated with an increase in the oxidative stress (Gella and Durany, 2009; Fawzy Fahim et al., 2018; Minaz et al., 2018; Rahman et al., 2018; Gomaa et al., 2019). Neuronal damage due to reactive oxygen species is known to cause memory dysfunction. Inhibition of sEH is known to reduce oxidative stress in various animal models (Goswami et al., 2016) including in STZ-induced memory dysfunction (Minaz et al., 2018). An increase in the level of MDA and nitrite, as well as a decrease in the levels of GSH, indicates an increase in oxidative stress. The anti-oxidant edaravone decreased the level of oxidative stress, and this also supports the hypothesis that oxidative stress is involved in diabetes-induced memory dysfunction. As expected, the sEHI TPPU decreased oxidative stress, and DHA potentiated the effect of TPPU presumably by the preservation of and an increase in epoxy-fatty acids such as the 19, 20-EDP from DHA.

Apart from oxidative stress, inflammation also induces neuronal damage (Aktas et al., 2007). Inflammation is also prominent in diabetes-associated memory impairment and cognition deficit (Cole et al., 2005; Chu et al., 2018; Tian et al., 2018). The increase in oxidative stress is commonly reported in Alzheimer’s disease (Gella and Durany, 2009). We also observed augmentation of inflammation in the brain of diabetic rats characterized by the increase in the levels of IL-1β and IL-6, but a decrease in the level of IL-10, in comparison to diabetic control rats. Treatment with edaravone or TPPU + DHA decreased the level of IL-6 in diabetic rats. Treatment with TPPU or TPPU + DHA increased the level of IL-10 in diabetic rats.

Association of memory loss with a parallel increase in the expression of APP in the brain of diabetic control mice suggests an association with Alzheimer’s disease in the diabetes model. Additionally, with the help of H&E staining, we showed an increase in the level of neuronal damage in diabetic rats, in comparison to healthy control rats. Edaravone (10 mg/kg) or TPPU decreased the level of APP and exerted neuroprotection. This suggests that the neuroprotective effect of TPPU at this dose is mediated by interfering with the expression of APP, in addition to decreasing neuroinflammation (level of IL-6) and oxidative stress (level of nitrite). Preventative treatment with TPPU (0.1–0.3 mg/kg) for eight weeks reduced the level of MDA but increased the level of glutathione in diabetic rats (Minaz et al., 2018). In this study, curative dosing of TPPU (1 mg/kg) did not alter the levels of MDA and GSH. Treatment with TPPU + DHA decreased the expression level of APP and protected against neuronal damage. We observed an increase in the activity of AChE in the hippocampus of diabetic rats in this study, but in our earlier study (Minaz et al., 2018), we have observed a decrease in the activity of AChE. This could be due to the difference in animal strains used (Wistar Albino vs. Sprague-Dawley), glycemic status (300 vs. 400 mg/dl), the age of animals used (3 vs. 1.5–2 months) and insulin supplementation (3 IU/kg s.c. twice a week vs. no supplementation).

Nevertheless, a decrease in the level of ACh (Hu et al., 2018) in the brain and an increase in the level of AChE in the hippocampus are documented in Alzheimer’s disease. Further, a reduction in the level of nicotinic receptors, one of the receptors of ACh is noticed in AD patients (Whitehouse et al., 1988; Maelicke and Albuquerque, 2000). Also, a reduction in the secretion of ACh and increase in metabolism by AChE is also implicated in AD (Maelicke and Albuquerque, 2000). Abnormality in choline transport and expression of cholinergic muscarinic receptor is also reported in AD (Terry and Buccafusco, 2003). Interestingly, splice variants of AChE are reported. Synaptic acetylcholinesterase (AChE-S) promotes AD via increasing amyloid fibril formation while “Readthrough” AChE-R protects neurons from Aβ. Surprisingly, an increase in the level of AChE-R mRNA and a decrease in the AChE-S mRNA was coupled with a reduction in AChE-R protein suggesting a complex role of AChE in AD (Berson et al., 2008). Treatment with edaravone (10 mg/kg), TPPU or TPPU + DHA decreased the level of AChE in the brain of diabetic rats.

Usefulness of DHA, a ω-3 fatty in various models of Alzheimer’s disease are reported (Calon et al., 2004, 2005; Lim et al., 2005; Ma et al., 2007; Kondo et al., 2013). Cole and his associates studied the effect of DHA on Alzheimer’s disease. Calon et al. demonstrated that depletion of dietary ω-3 fatty acids in mice overexpressing human Alzheimer’s disease gene APPswe (Tg2576) via treatment with safflower oil resulted in the activation of pro-apoptotic caspase-3 and *N*-methyl-D-aspartate receptor subunits (NR2A and NR2B) in cortex and hippocampus, and decreased the NR1 subunit in the hippocampus in addition to the decrease in the Ca^2+^/calmodulin-dependent protein kinase (CaMKII) in the cortex of transgenic animals. The adverse effects were minimal in wildtype mice fed with safflower oil. Supplementation of DHA minimized the changes mentioned above in the brain of transgenic mice (Carlon et al., 2005).

Further, Lim et al. (2005) demonstrated that DHA rich diet (0.6%) decreased the level of Aβ in the cortex of Tg2576 mice shown with ELISA. Image analysis using an antibody against Aβ further showed that a reduction in the level of plaque in the hippocampus and parietal cortex. DHA also decreased APP and its fragments. Kondo et al. (2013) reported that *in vitro* treatment with DHA to induce pluripotent stem cell-derived neurons and astrocytes from AD patients minimized various cell stresses including endoplasmic reticulum stress. Calon et al. (2004) demonstrated that reduction in dietary DHA to Tg2576 mice did not result in loss of neuron but increased caspase-cleaved actin (fractin) which is associated with loss of neuroprotective drebrin that has a role in neuronal growth. Tg2576 mice developed behavioral deficits with DHA depleted diet. Treatment with DHA protected the mouse with a decrease in the level of fractin in the brain and protected against behavioral deficits with a parallel increase in the phosphorylation of Bcl-2-associated death promoter (BAD) which has anti-apoptotic effect unlike BAD (Calon et al., 2004). Ma et al. (2007) reported that DHA increases the level of sortilin related receptor 1 (SORL1 or LR11) which is a receptor for apolipoprotein E and minimizes the neuronal sorting of APP to secretases that generate β-Amyloid (Aβ). DHA increased the level of LR11 in cultured primary rat neurons, frontal cortex of 22-monthC57B6/SJL and APP Tg2576 mice on DHA depleted diet and human neural SH-SY5Y cell lines. In the same study, the researchers showed that treatment with fish oil diet containing DHA and EPA to fructose-fed and insulin resistant 3–4-month-old rats increased the levels of LR11. This study demonstrated a mechanism of how DHA might regulate the level of Aβ because the level of LR11 is reported to be reduced in sporadic AD. Teng et al. (2015) reported that DHA prevents Aβ aggregation in APP/PS1 transgenic rat model of AD, but also suggested to combine DHA with other medications because DHA still increases less toxic fibrillar Aβ oligomers.

In a clinical trial, DHA was not able to slow the progression of AD (Quinn et al., 2010), which could be due to oxidation of DHA (Frautschy and Cole, 2011) and production of toxic levels of aldehydes and peroxides. Also, DHA’s epoxy metabolite is anti-inflammatory which is metabolized by sEH. Therefore, combining sEH with DHA would be more effective in controlling AD. Provided the published literature on the effect of DHA in AD, we have not included a DHA only group. Instead, according to our hypothesis, we tested if DHA increases the potency of sEHI. We observed that DHA + TPPU was more effective than only TPPU in alleviating inflammation and oxidative stress and augmented memory function. Thus, our data support the hypothesis that positive effects resulting from ω-3 supplementation of the diet are due, at least in part, to an increase in the bioactive epoxide metabolites of the ω-3 fatty acids.

Though we were supported by convincing data that sEH inhibitor TPPU ameliorates Alzheimer’s-like complications, TPPU should be further evaluated using Alzheimer’s disease-specific models. One of the limitations of this study is not evaluating the impact of STZ on the activity of sEH in the brain. Quantification of Aβ in the brain by western blot analysis and immunohistochemistry could have provided critical information. Documenting the body weight of animals and conducting glucose tolerance test and insulin tolerance test would have provided more insight into the complex biological pathology.

## Conclusion

In the present study, we demonstrated that sEHI alleviates cognitive and memory impairment associated with diabetes-induced Alzheimer-like complication. The positive effect of sEHI TPPU on memory was associated with a reduction in oxidative stress and inflammation in the brain with a parallel decrease in the mRNA level of APP and activity of AChE. The DHA potentiated the effect of TPPU. Edaravone at a higher dose also alleviated memory impairment via decreasing oxidative stress and inflammation.

## Author Contributions

RP, MA, SA, ML, and SG planned the experiments. RP, NB, KG, MA, and SA performed the experiments. RJ and SG analyzed the data. SG, KG, RJ, and BH wrote the manuscript. All the authors reviewed and approved the manuscript.

## Conflict of Interest Statement

The University of California, Davis holds multiple patents for the use of soluble epoxide hydrolase inhibitors in humans and companion animals. BH founded EicOsis LLC to facilitate the clinical use of soluble epoxide hydrolase inhibitors. The remaining authors declare that the research was conducted in the absence of any commercial or financial relationships that could be construed as a potential conflict of interest.
